# Cyclic and Acyclic Amine Oxide Alkyl Derivatives as Potential Adjuvants in Antimicrobial Chemotherapy against Methicillin-Resistant *Staphylococcus aureus* with an MDR Profile

**DOI:** 10.3390/antibiotics10080952

**Published:** 2021-08-06

**Authors:** Lorenza Fagnani, Lisaurora Nazzicone, Fabrizia Brisdelli, Luisa Giansanti, Sara Battista, Roberto Iorio, Sabrina Petricca, Gianfranco Amicosante, Mariagrazia Perilli, Giuseppe Celenza, Pierangelo Bellio

**Affiliations:** 1 Department of Biotechnological and Applied Clinical Sciences, University of L’Aquila, Via Vetoio 1, 67100 L’Aquila, Italy; lorenza.fagnani@graduate.univaq.it (L.F.); lisaurora.nazzicone@student.univaq.it (L.N.); fabrizia.brisdelli@univaq.it (F.B.); roberto.iorio@univaq.it (R.I.); sabrina.petricca@univaq.it (S.P.); gianfranco.amicosante@univaq.it (G.A.); mariagrazia.perilli@univaq.it (M.P.); pierangelo.bellio@univaq.it (P.B.); 2 Department of Physical and Chemical Sciences, University of L’Aquila, Via Vetoio 1, 67100 L’Aquila, Italy; luisa.giansanti@univaq.it (L.G.); sara.battista@univaq.it (S.B.)

**Keywords:** amphoteric surfactants, amine oxides, adjuvants, antibiotics, multidrug resistance, MRSA, cytotoxicity, checkerboard assay

## Abstract

The dramatic intensification of antimicrobial resistance occurrence in pathogenic bacteria concerns the global community. The revitalisation of inactive antibiotics is, at present, the only way to go through this health system crisis and the use of antimicrobial adjuvants is turning out the most promising approach. Due to their low toxicity, eco-friendly characteristics and antimicrobial activity, amphoteric surfactants are good candidates. This study investigated the adjuvant potentialities of commercial acyclic and newly cyclic *N*-oxide surfactants combined with therapeutically available antibiotics against MDR methicillin-resistant *Staphylococcus aureus* (MRSA). The safety profile of the new cyclic compounds, compared to commercial surfactants, was preliminarily assessed, evaluating the cytotoxicity on human peripheral mononuclear blood cells and the haemolysis in human red blood cells. The compounds show an efficacious antimicrobial activity strongly related to the length of the carbon atom chain. In drug–drug interaction assays, all surfactants act synergistically, restoring sensitivity to oxacillin in MRSA, with dodecyl acyclic and cyclic derivatives being the most effective. After evaluating the cytotoxicity and considering the antimicrobial action, the most promising compound is the L-prolinol amine-oxide C12NOX. These findings suggest that the combination of antibiotics with amphoteric surfactants is a valuable therapeutic option for topical infections sustained by multidrug-resistant *S. aureus*.

## 1. Introduction

In the last decade, the problem of antimicrobial resistance (AMR) has assumed the shape of a global emergency. As reported by the Centre for Disease Control and Prevention (CDC), the mortality rate per year in industrialised countries increases at an average of 23,000 deaths [1]. Six pathogens are mainly related to multi-resistance: *Enterococcus faecium*, *Staphylococcus aureus*, *Klebsiella pneumoniae*, *Acinetobacter baumannii*, *Pseudomonas aeruginosa* and *Enterobacter* spp. The acronym “ESKAPE”, from the first letter of the above-mentioned organisms, summarises their propensity to “escape” the biocidal action of antimicrobial agents [2,3]. *S. aureus* is one of the most important pathogenic bacteria, usually associated with nosocomial infections, expressing a multidrug resistance (MDR) profile, showing resistance to more than three classes of approved antibiotics. The increasing incidence of methicillin-resistant (MRSA), vancomycin-intermediate (VISA) and vancomycin-resistant (VRSA) *S. aureus* is a crucial issue, especially for community-acquired and hospital-acquired infections [4,5,6,7].

Although the 20th century is considered the “golden age” of antimicrobials, their misuse and sometimes abuse in clinical practice and veterinary medicine has also led to the selection of resistant bacterial strains [8,9], favouring the emergence of MDR strains. Despite the global antibiotic consumption growth of more than 65% between 2000 and 2015 worldwide, the number of deaths caused by infectious diseases [10,11] increased. In some cases, there is a total failure of antimicrobial therapies, and often effective molecules are no longer clinically available. From this point of view, the development of new antimicrobial drugs has also stalled. Hence the idea to develop molecules that can restore “old” antibiotic molecules or exert their action with mechanisms unrelated to the classic ones is attractive. The use of synthetic or natural drug combinations or adjuvants is beginning to open a new way to develop new antimicrobial solutions [7,12,13,14,15,16,17].

Surfactants play a key role in nanomedicine as building blocks of functional aggregates, as nanoparticle stabilisers and drug delivery systems, besides their great variety of properties (e.g., solubilisation, detergency, wetting and foaming ability) [18,19,20]. These aspects are strongly related to their molecular structure and composition. The charge of the polar headgroup and/or the length of the alkyl chain are essential to determine the surfactants’ behaviour in a solution; variations of the subtle balance between the hydrophobic and the hydrophilic portions of the molecules can affect the properties of both the monomers and the aggregates they form [21,22,23,24,25,26,27,28]. In particular, a pyrrolidine ring in the polar headgroup confers a lower degree of conformational freedom to the structure compared to the acyclic analogue [29]. This class of “soft” and environmentally friendly surfactants is very attractive in many fields, thanks to the possibility of modifying the length of the alkyl chain and the pyrrolidine skeleton [30,31,32]. *N*-oxides are very interesting among all the categories of surfactants because of their low or absent toxicity and irritative action, biodegradability, pH-sensitive aggregative behaviour and performances [33,34,35,36]. Generally, *N*-oxide surfactants can be easily prepared by the oxidation of the corresponding tertiary amine in the presence of hydrogen peroxide in an alcoholic medium. They are zwitterionic amphiphilic compounds at physiological pH, bearing a strong polar N–O bond with a high electron density on the oxygen atom [37]. In particular, these surfactants, coming from selective modification of their amphiphilic molecular structure, have opened the scenario to a plethora of systems [38,39,40,41,42,43,44,45], providing different superstructures by their self-assembly and co-assembly with other materials. They show multifunctional capabilities and are used in several industrial applications, such as washing-up products and laundry detergents, foaming and wetting agents, softeners and thickeners, besides being components of hair and body care products [46,47,48]. *N*-oxide surfactants can be an attractive solution as adjuvants in antimicrobial chemotherapy, to improve the activity of old antimicrobials, especially in drug formulations for external use, along with their propensity to be poorly or non-toxic.

**Figure 1:**
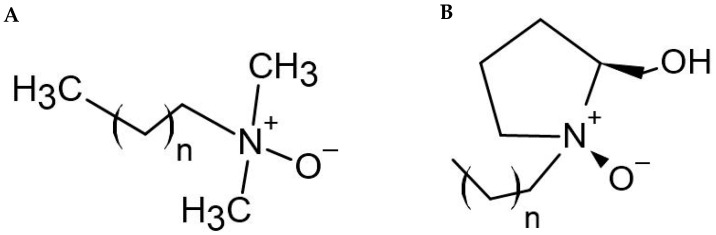
Structure of *N*-oxides. (**A**) Structures of commercial *N*-oxide alkyl derivatives: *n* = 10, LDAO; *n* = 12, TDAO. (**B**) Structures of L-prolinol *N*-oxide alkyl derivatives: *n* = 10, C12NOX; *n* = 12, C14NOX; *n* = 14, C16NOX.

In this study, we aimed to explore the potentialities of *N*-oxide-derived surfactants (Figure 1) as antibiotic adjuvants against MDR methicillin-resistant *S. aureus*. The antimicrobial activity of commercial acyclic *N*-oxide derivatives LDAO and TDAO, commonly found in many cleaning and personal care products, was compared with recently synthesised cyclic L-prolinol *N*-oxide analogues. The antimicrobial efficacy of the *N*-oxides was ascertained alone and in combination with therapeutically available antibiotics to determine the potential adjuvant properties. Moreover, a preliminary in vitro safety profile assessment of the cyclic L-prolinol *N*-oxide alkyl derivatives was determined and compared with commercial amphoteric surfactants.

## 2. Results

### 2.1. In Vitro Susceptibility Test

The compounds under examination were tested against 21 strains of methicillin-resistant *S. aureus* with an MDR profile (MIC for OXA ranging from 2 μg/mL to 256 μg/mL) to evaluate their antimicrobial activity. The MIC_50_ and MIC_90_ values for all compounds are reported in Table 1. The highest MIC value is related to LDAO (312.5 μM), while the lowest values are related to C14NOX and C16NOX, 4.88 μM and 2.44 μM, respectively.

### 2.2. Checkerboard Microdilution Assay

A two-dimensional checkerboard microdilution assay was used to evaluate the adjuvant effect of the detergents when combined with antibiotics against the reference strain *S. aureus* ATCC43300, which showed, in our experimental conditions, resistance to oxacillin (MIC = 32 μg/mL), clindamycin (MIC > 16,384 μg/mL), erythromycin (MIC > 2048 μg/mL) and gentamicin (MIC = 128 μg/mL). In addition, the antimicrobial susceptibility to detergents of the reference strain was also determined to arrange the conditions for the checkerboard assay. As shown in Table 2, the MIC values for the detergents were comparable to the MIC_50_ and MIC_90_ values previously determined for the clinical strains.

The data obtained from the checkerboard assays were analysed with a non-parametric interpretative model, the Loewe additivity-based model (Fractional Inhibitory Concentration Index, FICI), as reported in Table 3. Among the four antibiotics, no synergistic interaction was observed, except for the combination clindamycin-LDAO and oxacillin. On the other hand, the β-lactam oxacillin showed a synergistic effect with all detergents with an FICI ≤ 2. However, the most interesting results were observed when oxacillin is combined with LDAO and C12NOX, where the effective antibiotic concentration drops to 0.0625 µg/mL and 0.125 µg/mL, respectively.

### 2.3. Red Blood Cell Haemolysis and PBMC Cytotoxicity

A preliminary assessment of the safety profile of the L-prolinol *N*-oxide surfactants was accomplished by evaluating the haemolytic effect in red blood cells and the cytotoxicity in peripheral blood mononuclear cells. As described in Table 4, haemolysis occurred in a range between 19.5 μM for C16NOX and 625 μM for the dodecyl derivatives LDAO and C12NOX.

To verify the effect of the detergents on growth and viability in human cells, PBMCs were exposed to increasing concentrations of compounds for 48 h. The effects on cell growth were evaluated by estimating the *IC*_20_, *IC*_50_ and *IC*_80_ values, as reported in Table 4. Minor cytotoxicity was observed in the dodecyl derivatives LDAO and C12NOX with estimated *IC*_80_ values above 200 µM. For instance, LDAO showed inhibitory concentration values 2-fold higher than the equivalent tetradecyl derivative TDAO, and the C12NOX about 5–12-fold higher than the related derivatives with longer alkyl chains.

In red blood cell haemolysis experiments, the same trend can be observed for the amine oxide derivatives with a 12-atom carbon chain (Table 4). Indeed, both LDAO and C12NOX exhibit haemolytic activity at a concentration of 625 μM.

## 3. Discussion

*N*-alkyl-*N*,*N*-dimethylamine oxides are zwitterionic surfactants frequently used in many household cleaning products and personal care products. Amine oxides are generally considered environmentally eco-safe because they are fully biodegradable in aerobic and anaerobic conditions. Furthermore, from a human health safety profile point of view, it is known that amine oxides do not cause skin sensitisation, as well as no evidence of carcinogenic activity or reproductive and developmental toxicity has been reported [50,51,52].

This study aimed to investigate the potential antimicrobial activity and adjuvant antibiotic properties of L-prolinol *N*-oxide alkyl derivatives compared with the commercial *N*-oxide derivatives LDAO and TDAO, alone and in combination with therapeutically available antibiotics. Moreover, a preliminary safety profile was assessed by evaluating the induced haemolysis in red blood cells and cytotoxicity in peripheral blood mononuclear cells. The newly L-prolinol *N*-oxide derivatives differ from the “classical” alkyl dimethylamine oxide amphoteric surfactants for their peculiar headgroup, which originates by reduction of the carboxy group of the amino acid L-prolinol and oxidation of the tertiary pyrrolidinic amine [43]. Furthermore, the commercial surfactants LDAO and TDAO differ in the carbon chain length, C12 and C14, while the alkylic length of the newly synthesised L-prolinol *N*-oxide derivatives extends from 12 to 16 carbon atoms.

The compounds under examination were tested for their antimicrobial properties against twenty-one methicillin-resistant *S. aureus* clinical isolates with a multidrug resistant profile to determine their respective MIC values. Among the five formulations, the C14 and C16 alkyl L-prolinol derivatives show the greatest ability to inhibit microbial growth against all *S. aureus* strains (Table 1). This result can be elucidated by looking at the chemical features of the detergents and their composition. Subik J. et al. and further studies reported that the *N*-oxides’ antimicrobial activity is influenced by the carbon chain length [53,54,55]. Specifically, an alkyl chain length of approximately 15 carbon atoms was associated with maximum antimicrobial activity in dimethyl alkyl *N*-oxide surfactants [55]. Therefore, as the carbon chain length increases, the antimicrobial activity becomes more efficacious among surfactants bearing the same functional group, as can be observed for C16NOX, where the MIC_50_ and MIC_90_ are the lowest recorded, even though a plateauing effect is observed as the alkyl length increases (Table 1 and Table 2). One plausible explanation can be retrieved by looking at the critical micellar concentration (CMC) of these compounds. CMC describes the tendency of the surfactant monomer to form aggregates. For instance, it is reasonable to hypothesise that the low CMC values of the surfactant with the longest chain length might play a major role in determining its antimicrobial activity [43].

Since it is assumed that such compounds do not have specific targets, it is reasonable to suppose that they might be used as potential adjuvants to enhance the activity of the therapeutically available antibiotics against MDR organisms.

For this reason, *S. aureus* ATCC43300 was used as a model organism to verify a potential adjuvant effect in combination with those antibiotics whose efficacy is compromised by the presence of resistance. The MDR phenotype of *S. aureus* ATCC43300 confers resistance to various molecules belonging to four different classes of antibiotics, such as clindamycin (lincosamides), erythromycin (macrolides), gentamicin (aminoglycosides) and oxacillin (β-lactams). The potential synergistic effects of the surfactant–antibiotic combinations were assessed by checkerboard assay, and the data were analysed using the FICI model based on the Loewe additivity theory.

Among all tested antibiotics, clindamycin, erythromycin and gentamicin manifested no synergistic interaction, except for clindamycin in combination with LDAO, where a borderline value of FICI (0.5) was observed. On the contrary, both the commercial and new detergents exert a synergistic action, capable of enhancing the activity of the β-lactam oxacillin, verified by FICI values lower than 0.5. These data are worthy of attention since all surfactants reduced the MIC value of oxacillin below the breakpoint value (≤2 µg/mL), thus inducing a phenotypic reversion of resistance to oxacillin.

Unlike the other tested antibiotics, whose targets are located in the cytoplasmatic space of the bacterial cell, the targets of the β-lactam antibiotics are the penicillin-binding proteins (PBPs), enzymes with transpeptidase activity involved in the synthesis of peptidoglycan of the bacterial cell wall. PBPs are located outside the bacterial cell and are anchored to the external face of the bacterial plasma membrane through a 23-residue N-terminal transmembrane domain [56,57]. Resistance to methicillin in *Staphylococcus aureus* is prevalently mediated by the presence of a specific PBP, named PBP2′ or PBP2a, which is acquired by a mobile genetic element through horizontal gene transfer (SCC*mec*) [56,58]. PBP2a belongs to the subclass B1 of high-molecular-weight PBPs, characterised by a low β-lactam affinity [59]. However, bacteria gain resistance to lactam antibiotics by modifying their PBPs, thus preventing drug binding and, consequently, conferring resistance [56].

We can easily hypothesise that the tested zwitterionic surfactants most likely act at the level of the external structures of the cell, inducing a perturbation of the structure of the plasma membrane, which might affect the functionality of PBP2a.

The most effective interactions are between oxacillin and detergents with a 12-carbon atom chain length, regardless of the nature of the head. Indeed, thanks to LDAO and C12NOX, the MIC value for oxacillin drops below the breakpoint value, implying a 512-fold dose reduction in the antibiotic when combined with LDAO and a 256-fold dose reduction with C12NOX. Generally, the increase in the number of carbon atoms causes an increment in the antimicrobial activity when the compound acts alone [49,55], as demonstrated experimentally through the in vitro susceptibility test (Table 1 and Table 2). In reverse, we observe that the opposite happens with the combinations between the detergents and antibiotics; it is clear, in fact, that the best ability to hinder microbial growth is linked to detergents with a lower content of carbon atoms when combined with oxacillin. This result suggests that the surfactants support the action of the combined antibiotic with more efficacy in their monomeric form, also considering the previously estimated CMC values of cyclic amine *N*-oxides [43].

The idea to combine surfactants to enhance their broad-spectrum antimicrobial activity has been exploited in C31G, an equimolar combination of two synthetic C12 and C16 alkyl amine oxide and alkyl betaine [60,61], with antimicrobial and viricidal activity. Previous studies have demonstrated that C31G is effective against both Gram-positive and Gram-negative bacteria [60,61], fungi [62] and viruses [63]. Moreover, C31G possesses wound-healing and anti-inflammatory-related properties [64], and efficacious spermicidal activity [65]. Moreover, C31G was enrolled in a pivotal Phase III clinical trial to reduce the sexual transmission of the human immunodeficiency virus (a Phase II/III multicentre, randomized, double-masked study of the safety and contraceptive efficacy of C31G compared to Conceptrol^®^).

As stated above, global and national environmental agencies consider the safety profile of the *N*-alkyl-*N*,*N*-dimethylamine oxides sufficiently harmless from a human and environmental standpoint. In this respect, we intended to preliminary verify the safety profile of the L-prolinol *N*-oxide derivatives compared to the acyclic commercial surfactants. It was ascertained by evaluating the effect of the detergents on human peripheral blood mononuclear cell growth and viability. PBMCs were exposed to increasing concentrations of surfactants, and the cytotoxicity was evaluated by calculating the concentration inhibiting 20% (*IC*_20_), 50% (*IC*_50_) and 80% (*IC*_80_) of cell growth.

As reported in Table 4, commercially available surfactants are more tolerated than cyclic amine-oxides, although C12NOX shows growth-inhibitory concentrations comparable to those of LDAO and about two-fold above TDAO. If we compare the cytotoxicity inside the L-prolinol *N*-oxide series, we can observe a remarkable increase in toxicity as the alkyl moiety increases in carbon atoms. Indeed, the relationship between the carbon chain length and cytotoxicity has been observed by Catalone et al. in HeLa cells, where myristyl dimethyl amine oxide (16 atom carbon length) showed higher toxicity than TDAO [61]. Since this phenomenon is also observable between LDAO and TDAO, it is reasonable to affirm that the increase in the carbon chain length leads to higher toxicity. The same trend is observable in the cyclic *N*-oxide series, where the *IC*_80_ values for C14NOX and C16NOX are between 5- and 6-fold lower than the C12NOX.

However, it is noteworthy that the MIC_90_ values for *N*-oxide compounds with an alkyl chain longer than 12 carbon atoms, independently of the headgroup, are lower than the *IC*_50_ and *IC*_80_ values. It means that the efficacious antimicrobial dose is far from the cytotoxic dose. For instance, the MIC_90_ value for C16NOX is about 6-fold and 9-fold lower than the *IC*_50_ and *IC*_80_ values, while the MIC_90_ for LDAO is about 3-fold higher than the cytotoxic dose.

However, the most intriguing data are those obtained by the drug–drug combination assays. All detergents showed cytotoxicity values well above their effective concentrations when combined with oxacillin. It emerges that when C12NOX is combined with oxacillin, the detergent concentration is 9-fold lower than the *IC*_50_ value.

We must also consider that the C12NOX MIC_90_ value is 4-fold lower than the concentration causing the complete haemolysis of human red blood cells. Moreover, the observed synergistic effect with oxacillin leads to a C12NOX dose reduction of about 32-fold lower than the haemolysis concentration. Nevertheless, LDAO and C12NOX share the same haemolytic concentration value. Looking at the evaluated safety profile, these data are encouraging, especially if we consider that LDAO is widely used in many personal care products [27]. Another remarkable aspect is that the value of the concentration causing extensive red blood cell haemolysis for C12NOX is up to 32 times higher than the same L-prolinol *N*-oxides with a longer carbon chain. In this regard, it is plausible to assert that the length of the carbon chain, as in PBMCs, influences red blood cell haemolysis, probably because of its effect on CMC values. In general, the longer the alkyl chain the lower the CMC. Furthermore, it is clear that when the surfactants are organised in micelles, they exert their detergent effect (thus causing haemolysis in this case) with greater efficacy with respect to the same amphiphiles in their monomeric form. Therefore, the chain with 12 carbon atoms would seem to guarantee greater safety of the compound. Therefore, limited to the data obtained highlighting the absence of toxicity, we could assert, along with the other detergents already on the market, that C12NOX is a good candidate from a safety point of view, deserving further investigations.

Due to their low toxicity, low environmental impact [27] and high versatility, *N*-oxide-based surfactants are compounds of great interest. For instance, it is not by chance that they are employed in a wide variety of industrial applications and daily life products [43]. Of great significance is the ability of amphoteric surfactants to hinder cell growth, acting in a non-specific way. The lack of a specific target is of extreme importance since it would avoid the development of resistant phenotypes, opening the possibility of improving the activity of antimicrobials to which pharmacological resistance mechanisms may exist, such as oxacillin. Resistance to available antibiotics in pathogenic bacteria is currently a global challenge since the number of resistant strains to multiple classes of antibiotics has dramatically increased, spreading worldwide [66]. Nowadays, the new frontier of antimicrobial therapy is trying to revert antibacterial resistance using efficacious combinations of drugs to restore the activity of old and inactive molecules [14,15]. Therefore, antibiotic adjuvants in combination with available antibiotics are now being exploited to overcome the phenomenon of antimicrobial resistance. Antibiotic adjuvants, also known as ‘resistance breakers’ or ‘antibiotic potentiators’ [67,68,69,70,71,72], have no or poor antimicrobial activity, but when combined with antibiotics, they enhance the antimicrobial action of the drug [66]. Nowadays, resistance to amphoteric surfactants in pathogenic microorganisms has not been reported, and the use of these molecules can be exploited as adjuvants in antimicrobial therapy, specifically for topical use.

## 4. Materials and Methods

### 4.1. Organisms

Twenty methicillin-resistant *S. aureus* clinical strains with a multidrug-resistant profile (MDR), collected at the “San Salvatore” Hospital of L’Aquila, were used for the antimicrobial susceptibility test. In addition, a reference strain with a methicillin-resistant profile from the American Type Culture Collection *S. aureus* ATCC43300 was used to investigate the drug–drug interaction between the compounds and antibiotics. In addition to oxacillin, all these strains were resistant to clindamycin, erythromycin and gentamicin.

### 4.2. Antibiotics and Compounds

All the tested antibiotics, oxacillin, clindamycin, erythromycin and gentamicin, were from Sigma–Aldrich (Milan, Italy). *N*,*N*-Dimethyldodecan-1-amine *N*-oxide, also known as lauryldimethylamine *N*-oxide (LDAO) and *N*,*N*-Dimethyltetradecan-1-amine *N*-oxide, also known as myristyldimethylamine *N*-oxide (TDAO), were from Sigma-Aldrich (Milan, Italy). The L-prolinol amine oxide derivatives [(1*S*,2*S*)-1-dodecyl-1-oxo-1λ^5^-pyrrolidin-2-yl]methanol (C12NOX), [(1*S*,2*S*)-1-tetradecyl-1-oxo-1λ^5^-pyrrolidin-2-yl]methanol (C14NOX) and [(1*S*,2*S*)-1-hexadecyl-1-oxo-1λ^5^-pyrrolidin-2-yl]methanol (C16NOX) were synthesised as previously described [43].

### 4.3. In Vitro Susceptibility Test

The in vitro antimicrobial effect of the detergents against the organisms used in this study was ascertained by the microdilution method in 96-well microplates. The determination of the MIC values was done following the CLSI guidelines (CLSI, 2018). The microtiter plates were incubated for 18 h at 37 °C before MIC determination. A microplate reader iMark, BioRad (Milan, Italy), quantified at 595 nm the growth in each well. The minimum inhibitory concentration (MIC) for drugs and detergents was defined as the concentration of drug that reduced growth by 80% compared to that of organisms grown in the absence of a drug. The MIC value was determined as the median of at least three independent experiments. In addition, the MIC_50_ and MIC_90_ were also calculated as the lowest concentration of the compound at which the growth of 50% and 90% of the organisms were inhibited.

### 4.4. Checkerboard Microdilution Assay

The two-dimensional checkerboard microdilution assay using a 96-well microtitration plate was used to investigate the in vitro interactions between the antibiotics and detergents, as previously described by Segatore et al. [73,74]. The microtiter plates were incubated at 37 °C for 18 h. The growth in each well was quantified spectrophotometrically at 595 nm by a microplate reader. The percentage of growth in each well was calculated as follows:

$$\frac{OD_{drug\;combination\;well\;}-\;OD_{background}}{OD_{drug\;free\;well}-OD_{background}}\;\times \;100$$

where the background is derived from the microorganism-free plates. All experiments were performed in triplicate.

### 4.5. Drug–Drug Interaction Model Analysis

The data acquired from the checkerboard assay were examined by a non-parametric interpretative model based on the Loewe additivity model (Fractional Inhibitory Concentration Index, FICI), as previously described [74,75].

### 4.6. Human Peripheral Blood Red Cells and Mononuclear Cells (PBMCs) Isolation

Peripheral blood mononuclear cells (PBMCs) obtained from heparinised whole blood of healthy adults were isolated by density gradient centrifugation using Histopaque-1077 (Sigma–Aldrich, St. Louis, MO, USA). The PBMCs were collected, washed twice and resuspended in RPMI 1640 medium supplemented with 10% foetal bovine serum, 2 mM glutamine, 100 U/mL penicillin and 100 µg/mL streptomycin.

### 4.7. Human Red Blood Cell Haemolytic Assay

Haemolytic activity of detergents on human erythrocytes was determined as previously described [73]. Briefly, after washing three times with phosphate-buffered saline (PBS 5 mM phosphate buffer containing 150 mM NaCl, pH 7.4), red blood cells were isolated from heparinised human blood by centrifugation. Erythrocytes were diluted in PBS saline to reach an absorbance of 1.0 at 655 nm. In a sterile 96-well microtiter plate, already containing 100 μL of the diluted compound, an aliquot of 100 μL of erythrocytes was added to reach a final volume of 200 μL. Complete haemolysis (100% haemolysis) was ascertained by preparing wells with PBS saline containing 0.2% Triton X-100 and red blood cells. The absence of haemolysis (0% haemolysis) was verified by preparing wells with PBS saline and erythrocytes. The microtitration plate was incubated at 37 °C with gentle mixing for 20 min. The haemolysis was quantified spectrophotometrically at 655 nm by a microplate reader (iMark, BioRad, Milan, Italy). The haemolysis percentage was obtained using the following equation:

$$hemolysis\;\left(\%\right)=100-\frac{OD_{655\;nm\;well\;containing\;detergent}-\;OD_{655\;nm\;0\%\;hemolysis}}{OD_{655\;nm\;100\%\;hemolysis\;}-\;OD_{655\;nm\;0\%\;hemolysis}}\;\times \;100$$

All experiments were done in triplicate.

### 4.8. PBMC Cytotoxicity Assay

The effects of the detergents on peripheral blood mononuclear cells (PBMCs) viability were evaluated by the MTT colourimetric method that quantifies the cellular metabolic activity [75]. PBMCs, seeded in 96-well plates at a density of 1 × 10^6^ cells/mL (1 × 10^5^ cells/well), were treated with increasing concentrations of compounds (LDAO and C12NOX from 75 to 300 µM; TDAO from 25 to 150 µM; C14NOX and C16NOX from 5 to 50 µM) for 48 h, at 37 °C in a humidified atmosphere of 5% CO_2_.

At the end of treatment, the MTT solution was added to each well at a concentration of 0.5 mg/mL and cells were incubated at 37 °C for a further 4 h. Then, the MTT-formazan crystals were dissolved in acidified isopropanol (0.04 M HCl in isopropanol), and the absorbance at 570 nm was determined using a microplate reader (Biorad, Model 550).

The percentage of cell survival was obtained by comparing the absorbance of the treated groups with that of the untreated cells, the viability of which is taken as 100%. Experimental data for the calculation of the *IC*_20_ and *IC*_50_ were analysed using OriginPro 2018 software.

## Figures and Tables

**Table 1:** MIC_50_ and MIC_90_ calculated for 21 *S. aureus* clinical isolate strains.

Compounds (Carbon Chain Length)	MIC_50_ (µM) (Range)	MIC_90_ (µM)	CMC * (M)
LDAO (12)	312.50 (39.06–625.00)	625.00	^a^ 1.7 × 10^−3^
TDAO (14)	19.53 (19.53–39.06)	39.06	^a^ 2.7 × 10^−4^
C12NOX (12)	156.25 (9.77–156.25)	156.25	^b^ 1.4 × 10^−5^
C14NOX (14)	4.88 (1.22–19.53)	9.77	^b^ 5.8 × 10^−6^
C16NOX (16)	2.44 (0.61–19.53)	4.88	^b^ 1.5 × 10^−6^

* Critical micelle concentration; ^a^ Reference [43]; ^b^ Reference [49].

**Table 2:** MIC values of the detergents calculated for *S. aureus* ATCC43300.

Compounds (Carbon Chain Length)	Median MIC (µM) (Range)
LDAO (12)	312.50
TDAO (14)	39.06
C12NOX (12)	78.13 (78.13–156.25)
C14NOX (14)	19.53 (9.77–19.53)
C16NOX (16)	19.53 (9.77–19.53)

**Table 3:** Results from the in vitro interaction experiments between detergents and antibiotics against *S. aureus* ATCC43300 determined by non-parametric FICI.

				Effective Combination	
Compounds (Carbon Chain Length)	Antibiotic	FICI_min_	INT *^a^*	Antibiotic (µg/mL)	Detergents (µM)
LDAO (12)	clindamycin	0.5	SYN	16	156.25
	erythromycin	0.625	IND		
	gentamicin	1	IND		
	oxacillin	0.252	SYN	0.0625	78.13
TDAO (14)	clindamycin	1	IND		
	erythromycin	1	IND		
	gentamicin	0.531	IND		
	oxacillin	0.313	SYN	2	9.77
C12NOX (12)	clindamycin	1	IND		
	erythromycin	1	IND		
	gentamicin	1	IND		
	oxacillin	0.254	SYN	0.125	19.53
C14NOX (14)	clindamycin	1	IND		
	erythromycin	1	IND		
	gentamicin	1	IND		
	oxacillin	0.5	SYN	2	4.88
C16NOX (16)	clindamycin	1	IND		
	erythromycin	1	IND		
	gentamicin	1	IND		
	oxacillin	0.5	SYN	2	4.88

*^a^* INT, interpretation; IND, indifference; SYN, synergism; ANT, antagonism. Synergism was defined as an FICI of ≤0.5, antagonism was defined as an FICI of >4, and indifference was defined as an FICI >0.5 and ≤4.

**Table 4:** Preliminary assessment of the safety profile of L-prolinol *N*-oxide compounds compared to *N*-oxide detergents. It includes the calculated values of *IC*_20_*, IC*_50_ and *IC*_80_ from dose–response experiments in human peripheral blood mononuclear cells (PBMCs) and the concentration of the detergents causing 100% of human red blood cell (RBC) haemolysis.

Compounds (Carbon Chain Length)	*IC*_20_ ± SEM *^a^*(µM)	*IC*_50_ ± SEM *^a^* (µM)	*IC*_80_ ± SEM *^a^* (µM)	Haemolysis * (µM)
LDAO (12)	192.9 ± 8.6	220.1 ± 6.6	250.0 ± 11.1	625.0
TDAO (14)	100.3 ± 2.3	113.6 ± 1.6	128.7 ± 2.9	78.2
C12NOX (12)	154.3 ± 4.1	177.3 ± 4.7	203.6 ± 5.4	625.0
C14NOX (14)	12.5 ± 1.9	20.8 ± 1.4	34.7 ± 3.3	78.2
C16NOX (16)	18.3 ± 2.8	27.6 ± 2.3	41.7 ± 5.1	19.5

*^a^* Error is expressed as ± standard error (SEM) of the mean of three independent experiments. * Intended as the median concentration value, obtained from three independent experiments, causing 100% of human red blood cell haemolysis in a double-serial dilution assay.

## Data Availability

Data is contained within this article.
